# Modification of astrocytic Cx43 hemichannel activity in animal models of AD: modulation by adenosine A_2A_ receptors

**DOI:** 10.1007/s00018-023-04983-6

**Published:** 2023-10-29

**Authors:** Daniela Madeira, Joana Domingues, Cátia R. Lopes, Paula M. Canas, Rodrigo A. Cunha, Paula Agostinho

**Affiliations:** 1 Faculty of Medicine, University of Coimbra (FMUC), Coimbra, Portugal; 2 Center for Neuroscience and Cell Biology (CNC), University of Coimbra, Rua Larga, Polo I FMUC, First Floor, 3004-504 Coimbra, Portugal

**Keywords:** Alzheimer’s disease, Astrocytes, Adenosine A_2A_ receptors, Hemichannels, Connexin 43

## Abstract

Increasing evidence implicates astrocytic dysfunction in Alzheimer’s disease (AD), a neurodegenerative disorder characterised by progressive cognitive loss. The accumulation of amyloid-β (Aβ) plaques is a histopathological hallmark of AD and associated with increased astrocyte reactivity. In APP/PS1 mice modelling established AD (9 months), we now show an altered astrocytic morphology and enhanced activity of astrocytic hemichannels, mainly composed by connexin 43 (Cx43). Hemichannel activity in hippocampal astrocytes is also increased in two models of early AD: (1) mice with intracerebroventricular (icv) administration of Aβ_1-42_, and (2) hippocampal slices superfused with Aβ_1-42_ peptides. In hippocampal gliosomes of APP/PS1 mice, Cx43 levels were increased, whereas mice administered icv with Aβ_1-42_ only displayed increased Cx43 phosphorylation levels. This suggests that hemichannel activity might be differentially modulated throughout AD progression. Additionally, we tested if adenosine A_2A_ receptor (A_2A_R) blockade reversed alterations of astrocytic hemichannel activity and found that the pharmacological blockade or genetic silencing (global and astrocytic) of A_2A_R prevented Aβ-induced hemichannel dysregulation in hippocampal slices, although A_2A_R genetic silencing increased the activity of astroglial hemichannels in control conditions. In primary cultures of astrocytes, A_2A_R-related protective effect was shown to occur through a protein kinase C (PKC) pathway. Our results indicate that the dysfunction of hemichannel activity in hippocampal astrocytes is an early event in AD, which is modulated by A_2A_R.

**Supplementary Information:**

The online version contains supplementary material available at 10.1007/s00018-023-04983-6.

## Introduction

Alzheimer’s disease (AD) is a neurodegenerative disorder characterised by a progressive decline of cognitive functions, namely learning and memory, which is linked with an abnormal accumulation of amyloid-β peptides (Aβ). Although the presence of Aβ plaques is a major histopathological hallmark of established AD, it is considered that the extracellular accumulation of soluble Aβ oligomers is a causative agent of neurodegeneration, mainly of synaptic deterioration that is usually associated with the first signs of memory impairments preceding the formation of Aβ deposits that are characteristic of early AD (reviewed in [1]). The hippocampus is particularly affected in early stages of AD, undergoing structural and functional changes typified by alterations of synaptic plasticity that are considered the neurophysiological basis of learning and memory encoding (reviewed in [2]). Astrocytes are glial cells with multiple processes that establish contact with 60–70% of hippocampal synapses and regulate synaptic plasticity, mainly through their capacity to release gliotransmitters, such as ATP and glutamate, and to uptake glutamate and GABA from the synaptic cleft [3–5]. Accordingly, increasing evidence supports a role of astrocytes in AD onset and progression, as heralded by several morphological and molecular changes of astrocytes in AD mouse models (e.g. APP/PS1) and in human patients [6, 7] as well as by the link between Aβ plaques formation and changes in astrocytes morphology and activity [8, 9].

Astrocytic responses depend on the functions of connexins (Cx), which are proteins forming gap junction channels and hemichannels that allow inter-cellular fluxes of ions and small molecules, such as IP_3_, ATP, glutamate, and energy metabolites (reviewed in [10, 11]). Astrocytic hemichannels are mainly composed of hexamers of Cx43 and Cx30, which accumulate in astrocyte processes allowing the release of gliotransmitter and ion fluxes that can regulate hippocampal synaptic transmission and plasticity, and consequently memory [12, 13]. Astrocytic Cx43 levels are upregulated in animal models of AD, namely in transgenic APP/PS1 and 5xFAD mice [14–16]. Moreover, there are evidences of dysfunctional astrocytic hemichannels in animal models of AD that do not exhibit alterations on gap junction astrocytic communication [17–19]. Furthermore, in adult APP/PS1 mice with 8–18 months of age, it was reported that activated microglial cells did not contribute to the elevated connexin immunoreactivity that was concentrated in astroglial processes infiltrating the amyloid plaques [14], implying that the AD-related alteration of connexins hemichannels is intrinsic to astrocytes.

Our group previously showed that cultured astrocytes exposed to Aβ peptides display an enhancement of Cx43 hemichannels and that adenosine A_2A_ receptors (A_2A_R), which are closely associated with Cx43, regulate the levels and activity of hemichannels [20]. A_2A_R modulate key astrocytic functions that are affected by Aβ exposure, such as glutamate uptake [21], ATP release and hemichannel activity [20], and Ca^2+^ signalling [22], and we recently showed that the genetic deletion of astrocytic A_2A_R in the hippocampus of adult mice impairs synaptic plasticity and cause memory deficits [23]. Moreover, several studies support the idea that A_2A_R are a promising target to manage AD, since the pharmacological or genetic blockade of A_2A_R confers neuroprotection [24–28]. Likewise, as occurs for Cx43, astrocytic A_2A_R are increased in the hippocampus of mice exposed intra-cerebro-ventricularly to Aβ [21], mice expressing human amyloid precursor protein [29], 3xTg-AD mice [28] and APP/PS1 mice [26] as well as in AD patients [29]. However, it remains to be clarified if the activity of Cx43 hemichannels is modified in early AD and whether A_2A_R regulate the activity of hemichannels in hippocampal astrocytes. Thus, in the present study, we resorted to in vivo and ex vivo models of early AD to investigate alterations in astrocytic hemichannel activity in hippocampal slices and tested if A_2A_R modulate Cx43 hemichannel activity under physiological and AD-like conditions.

## Materials and methods

### Animals and surgeries

Animals were kept with food and water available ad libitum under a controlled environment (23 ± 2 °C, 50 ± 10% relative humidity and 12 h light/dark cycle). Mice were handled following the “3R” principles, and all experiments were approved by the Ethical Committee of the Center for Neuroscience and Cell Biology of the University of Coimbra (ORBEA_300_2021/24092021) and certified by *Direção Geral de Alimentação e Veterinária* (DGAV; Portuguese National Authority for Animal Health and Well-Being, 0421/000/000/2021).

A line of double transgenic mice, APPswe/PS1dE9 (APP/PS1), with B6C3F1/J genetic background (Jackson Laboratory, Bar Harbor, Maine, strain B6;C3-Tg(APPswe,PSEN1dE9)85Dbo/Mmjax RRID:MMRRC_034832-JAX) and wild type (WT) littermates were used. We chose to use male mice, to avoid estrous cycle influences, with 9 months old, as the deficits of reference memory and of synaptic plasticity are already evident in both males and females at this age [25, 30]. We also used C57Bl/6 male mice obtained from Charles River Laboratories Barcelona, Spain (MGI catalogue number (Cat#) 5811150, Research Resource Identifier, RRID:MGI:5811150), global A_2A_R knockout (GbA_2A_RKO) and forebrain neuron conditional knockout mice (FbA_2A_RKO) with C57Bl/6 genetic background that were generated by Jiang-Fan Chen (Boston University School of Medicine, MA). The selective deletion of A_2A_R in astrocytes was accomplished in A_2A_R floxed (A_2A_^flox/flox^) mice through the Cre-lox method. Briefly, an AAV5-GFAP-GFP-CRE viral construct (1 µL from 4.8 × 10^12^ particles/mL, obtained from Vector Core, University of North Carolina, USA) was bilaterally administrated into the CA1 region of the dorsal hippocampus (GFAP-CRE-A_2A_R mice), whereas control (GFAP-CTR) mice received a similar construct without Cre recombinase, AAV5-GFAP-eGFP (Vector Core, University of North Carolina, USA), as previously described [23].

Some C57Bl/6 mice (5 mice) were subjected to an intra-cerebro-ventricular (icv) administration of the synthetic peptide Aβ_1-42_ (Bachem), carried out as previously described [24, 31]. Aβ_1-42_ was dissolved in sterile water to obtain a solution mostly composed of soluble monomers and low molecular weight oligomers (2.25 mg/mL) and 4 μL of Aβ_1-42_ (2 nmol) or water (vehicle) were administered icv, which translates into 5–30 pmol levels of Aβ_1-42_ within the hippocampus [24]. This Aβ_1-42_-icv model recapitulates two main features of early AD, namely the impairment of reference memory and of synaptic function, as we previous reported [24, 31].

### Morris water maze

The Morris water maze test was performed as previously described [32] in order to evaluate hippocampal-dependent spatial learning and memory. The test was done using a 105 cm diameter circular pool filled with water opacified with non-toxic white paint. A platform was submerged 1 cm underneath water surface. The acquisition training phase consisted of four swimming trials of 60 s per day with a 20 min inter-trial interval. In each trial, mice were placed in the pool from a different drop location and given 60 s to find the location of the hidden platform. To complete the trial, the animals must remain 10 s on the platform. This stage was completed when the average of escape latency from the four drop points reached 20 s in WT mice. It was then followed by a retention/probe trial in which the platform was removed and mice were placed on the pool from a random drop location and allowed to swim freely for 60 s. The number of crossings of the hidden platform location was recorded with Any-Maze version 4.99 tracking software (Stoelting, Wood Dale, USA, RRID:SCR_014289). The strategies used by mice to find the platform location in the retention trial were analysed offline and classified as hippocampus-dependent (allocentric) or hippocampus-independent (egocentric), as previously described [33].

### Pharmacological treatments in hippocampal slices

Mice were sacrificed by decapitation after deep anaesthesia with halothane. The brain was removed and the hippocampus dissected in ice-cold, oxygenated (95% O_2_ and 5% CO_2_) artificial cerebrospinal fluid solution (aCSF, composition: 124 mM NaCl, 3 mM KCl, 1.25 mM NaH_2_PO_4_, 26 mM NaHCO_3_, 10 mM glucose, 1 mM MgSO_4_, and 2 mM CaCl_2_; pH 7.4). Transversal hippocampal slices (400 μm-thick) were obtained with a McIlwain tissue chopper, and then allowed to recover functional and energetically in gassed ACSF for 90 min at 32 °C.

Control hippocampal slices (CTR) were only superfused with aCSF. For Aβ_1-42_ challenge, hippocampal slices were superfused with Aβ_1-42_ (50 nM in aCSF) for 60 min, a concentration and time of peptide exposure that we previously showed to affect synaptic plasticity (see [34]), before assaying hemichannel activity. Aβ_1-42_ solution contained mainly soluble monomers and oligomers, as previously reported by us [21, 24]. When testing the effect of A_2A_R, slices were incubated for 30 min with the A_2A_R selective antagonist, SCH58261, [(2-(2-furanyl)-7-(2-phenylethyl)-7H-pyrazolo[4,3-e][1,2,4]triazolo[1,5-c]pyrimidin-5-amine, 50 nM in aCSF, Tocris Bioscience] prior to the challenge with Aβ_1-42_, and continuously superfused with SCH58261 during the exposure to Aβ_1-42_. In experiments with APP/PS1 mice, hippocampal slices were superfused with Gap19 (250 μM in aCSF; Tocris Bioscience) for 90 min before assaying hemichannel activity.

### Ethidium bromide uptake assay and immunolabelling of astrocytes

The activity of hemichannels was carried out with an ethidium bromide (EtBr) assay as previously described [35, 36]. Briefly, after the pharmacological treatments, hippocampal slices were incubated with EtBr (20 μM; Sigma-Aldrich) in aCSF solution in an oxygenated chamber for 5 min at room temperature (RT) and rinsed with gassed aCSF for 15 min to stop EtBr uptake. Hippocampal slices were then fixed by immersion overnight at 4 °C in a 4% paraformaldehyde (PFA) solution in PBS (137 mM NaCl, 2.7 mM KCl, 10 mM Na_2_HPO_4_ and 1.9 mM KH_2_PO_4_, pH 7.4) and then rinsed with a glycine solution (0.5 M) for 5 min to block unreacted aldehydes that produce background signals. This step was followed by several washes with PBS. Slices were protected from light throughout the entire procedure and were further processed for GFAP immunolabelling, as previously described [23]. Briefly, hippocampal slices were permeabilized for 30 min with 0.1% Triton X-100 in PBS and then with blocking solution (10% horse serum, 0.1% Triton X-100 in PBS) for 2 h. Slices were then incubated for 2 days at 4 °C with the primary antibody, rabbit anti-GFAP (1:1000, Millipore Cat# AB5804, RRID:AB_2109645) diluted in blocking solution. After washing, slices were incubated first with a blocking solution for 30 min and then with a donkey anti-rabbit 488 (1:1000, Thermo Fisher Scientific Cat# A-21206, RRID:AB_2535792) secondary antibody for 4 h at RT. In the case of hippocampal slices obtained from GFAP-CRE-A_2A_R mice, we also used as a primary antibody goat anti-GFP (1:500, Abcam Cat# ab6673, RRID:AB_305643) and anti-goat 488 (1:1000, Molecular Probes Cat# A-11055, RRID:AB_2534102) and an anti-rabbit 647 (1:1000, Molecular Probes Cat# A-21245, RRID:AB_141775) as secondary antibodies. Hippocampal slices were then washed three times with PBS during 10 min, incubated with Hoechst33258 (1:2000, Sigma Aldrich) for 30 min at RT to stain the nuclei, washed again three times during 10 min with PBS and mounted onto Ibidi μ-slide 8 well treated using Fluoromount Aqueous Mounting Medium (Sigma-Aldrich). Hippocampal slices were visualised with a LSM 710 confocal inverted microscope (Zeiss, RRID:SCR_018063) with a 40× objective (Plan-Apochromat 40×/1.4 Oil DIC M27 objective) and Z-stacks were acquired using Black Zen software (RRID:SCR_018163).

Analysis of EtBr fluorescence signal in astrocytes involved the processing of the images obtained by confocal microscopy through deconvolution using Huygens software (RRID:SCR_014237) to reduce out-of-focus information, thus increasing spatial resolution through a classic maximum likelihood estimation (CMLE) algorithm. Then, a normalisation operation was conducted in the GFAP channel in order to improve visualisation of GFAP^+^ cells with Imaris software (RRID:SCR_007370), which was used in the subsequent processing stages and analysis. The next step was a nuclei segmentation through creation of surfaces in the nuclei channel. Astrocyte nuclei were manually selected according to the following criteria: (i) cells positive for GFAP which comprehend a single nucleus enwrapped by a GFAP immuno-labelled structure, (ii) nucleus not truncated and (iii) nucleus exhibiting EtBr fluorescence above background. An additional criterion was considered when analysing hippocampal slices from GFAP-CRE-A_2A_R mice: only GFAP-positive cells with GFP immuno-labelling were evaluated. EtBr uptake was quantified as a ratio between EtBr fluorescence intensity (in arbitrary units, au) and nucleus volume (µm^3^) for each astrocyte (i.e. GFAP^+^ cell); we then calculated, for each experimental condition, the mean of EtBr uptake normalised (ratio) to the corresponding control condition. It should be mentioned that all GFAP^+^ cells take up EtBr and that we checked for differences in the volume of astrocyte nuclei amongst the different experimental conditions analysed and no statistically significant differences were found (data not shown).

### Aβ immunohistochemistry and thioflavin-S staining

APP/PS1 and WT mice were transcardially perfused with ice-cold PBS followed by 4% PFA in PBS. Brains were removed, post-fixed for 24 h in PFA, dehydrated in 30% sucrose solution for 72 h at 4 °C and cryopreserved at −80 °C. Coronal brain sections (30 μm) were obtained using a cryostat (CryoStar NX50, ThermoScientific, RRIDD:SCR_022732) for Aβ immunolabelling and thioflavin-S staining following a previously described protocol with minor modifications [37]. First, antigen retrieval was performed through the incubation of air-dried sections in citrate buffer (10 mM sodium citrate tribasic dehydrate, 0.05% Tween 20, pH 6.0) at 95 °C for 15 min. Then, sections were rinsed three times with PBS for 5 min, permeabilized with a 0.2% Tween 20 solution in PBS for 15 min and non-specific binding blocked by incubation of 1% bovine serum albumin (BSA) 0.05% Tween 20 in PBS for 30 min. Sections were incubated overnight in an airtight humidity chamber with the mouse anti-β-amyloid (βA) primary antibody (1:250, Covance Cat# SIG-39320, RRID:AB_662798) in blocking solution. Sections were washed again three times in PBS for 5 min prior to incubation for 2 h with the secondary anti-mouse Alexa594 antibody (1:1000, Molecular Probes Cat# A-21203, RRID:AB_141633). Following three rinses with PBS for 5 min, sections were stained with 1% thioflavin-S solution for 10 min, differentiated in 70% ethanol, rinsed thrice with water, mounted with DAKO mounting medium and cover-slipped. Finally, hippocampal sections were visualised by fluorescence microscopy (Zeiss, Axio Imager Z2 microscope, RRID:SCR_018856) and the pictures were captured using the AxioVision Imaging System (RRID:SCR_002677 version 4.8).

### Immunohistochemistry in brain slices

Free floating coronal brain sections (30 μm-thick) from APP/PS1 and WT mice were obtained as described above. For GFAP immunolabelling, brain sections were rinsed with PBS, incubated with permeabilization solution (0.1% Triton-X100 in PBS) for 15 min and then with blocking solution (10% horse serum + 0.1% Triton-X100 solution in PBS) for 2 h. Afterwards, sections were incubated with the rabbit anti-GFAP (1:1000, Millipore Cat# AB5804, RRID:AB_2109645) primary antibody overnight at 4 °C. Sections were then washed with PBS, further incubated with anti-rabbit Alexa594 (1:1000, Thermo Fisher Scientific Cat# A-21207, RRID:AB_141637) for 2 h at RT and washed again with PBS. Nuclei were stained with DAPI (1:5000, Invitrogen) for 10 min at RT. Following rinsing with PBS, sections were mounted onto gelatin-coated slides using ProLong™ Antifade mounting medium (Cell Signaling Technology). Z-stack images were captured with intervals of 0.5 μm with a LSM 710 confocal inverted microscope (Zeiss, RRID:SCR_018063) with a 63 × objective (Plan-Apochromat 63x/1.40 Oil DIC M27) for the tridimensional reconstruction of the astrocytic structure.

### 3D reconstruction of astrocytes

The morphology of astrocytes was studied as previously described [23, 38, 39] by tridimensional reconstruction of astrocytic structures using an open access tool, Simple Neurite Tracer (SNT, RRID:SCR_016566) plugin available in Fiji-ImageJ software. The tridimensional reconstruction of astrocytic processes within the *stratum radiatum* of the CA1 subregion of the dorsal hippocampus was carried out using Z-stack images as reported above. Astrocytes were selected for the tridimensional reconstruction of arbors of astrocytic processes according with the following criteria: (i) a single nucleus enwrapped by a GFAP-immunolabelled structure, (ii) the main structure of the astrocyte did not present truncated processes and (iii) reconstruction was carried out in the first five astrocytes fulfilling the previously mentioned criteria in each animal of both genotypes. The morphometric analysis of astrocytic arbor complexity was performed by quantifying the number of processes, their total length and the number of intersections with concentric spheres starting at the centre of the soma with intervals of 4 μm (Sholl analysis).

### Preparation of gliosomes

Gliosomes were obtained from hippocampal tissue through a discontinuous Percoll gradient as previously described [40, 41]. Briefly, hippocampal tissue was homogenised in ice-cold isolation sucrose solution (0.25 M sucrose, 10 mM HEPES, pH 7.4 at 4 °C) using a glass-Teflon tissue grinder. Nuclei and debris were removed by centrifugation (1000*g*, 5 min at 4 °C) and the supernatant was carefully placed on top of the discontinuous gradient composed by 23, 10, 6 and 2% v/v of Percoll in a sucrose solution (0.32 M sucrose, 1 mM EDTA, pH 7.4 at 4 °C), which was stratified by centrifugation (31,000*g* for 5 min at 4 °C), turning off the centrifuge brake for the last 2000*g* to avoid a sudden stop. Gliosomes were collected between the 2% and 6% v/v Percoll layers, whereas synaptosomes (purified synapses) were collected in the interface between the 23% and 10% of Percoll layers. Each fraction was washed with isotonic physiological solution 140 mM NaCl, 5 mM KCl, 5 mM NaHCO_3_, 1.2 mM NaH_2_PO_4_, 1 mM MgCl_2_, 10 mM glucose and 10 mM HEPES, pH 7.4 at 4 °C) and further centrifuged (30,000*g* for 20 min at 4 °C). Pellets were washed again in isotonic physiological solution and centrifuged (22,000*g* for 20 min at 4 °C), the supernatant was discarded and the pellets solubilised in RIPA lysis buffer supplemented with 1 mM dithiothreitol (Sigma Aldrich), 1 mM phenylmethylsulfonyl fluoride (PMSF, Sigma Aldrich), 0.001% of a protease inhibitor cocktail (CLAP; Sigma Aldrich) and phosphatases inhibitor cocktail phosphoSTOP (Roche). The comparative analysis of gliosomes and synaptosomes by Western blot allowed confirming the enrichment of our gliosomal preparation in proteins found in perisynaptic astrocytic processes, such as Cx43 and glutamate transporters (GLT-1 and GLAST), as can be observed in Figure S1, the gliosomes preparation of adult wild-type mice had an enrichment not only in GFAP levels, but also of Cx43, GLT-1 and GLAST, as compared with synaptosomal preparations that were enriched in synaptophysin, a widely used synaptic marker.

### Western blot

Western blot was performed as previously described [20]. The amount of protein samples used to quantify Cx43 and phosho-Cx43 at Ser368 was 3 and 30 µg, respectively; the amount of protein samples used to quantify other proteins was as follows: 3 µg GFAP, 20 µg for GLT-1 and GLAST and 10 µg for synaptophysin. Membranes were probed with rabbit antibodies against Cx43 (1:8000, Sigma-Aldrich Cat# C6219, RRID:AB_476857), anti-phospho-Cx43-Ser368 (1:1000, Cell Signaling Technology Cat# 3511, RRID:AB_2110169), GLT-1 (1:1000 ThermoFisher Scientific PA5-17099 RRID:AB_10978571) GLAST (1:1000 Abcam Ab416 RRID:AB_304334), GFAP (1:20,000, Millipore Ab5804 RRID:AB_2109645) or with mouse anti-synaptophysin antibody (1:20,000 Sigma S5768, RRID:AB_477523). These primary antibodies were diluted in Tris-buffered saline (137 mM NaCl, 20 mM Tris, pH 7.6) containing 0.1% Tween 20 (TBS-T) and 5% non-fat dry milk or 3% BSA overnight at 4 °C. After washing with TBS-T, membranes were incubated with IgG secondary antibodies (anti-rabbit, 1:10,000, RRID: AB_228338; anti-mouse 1:10,000, RRID:AB_228302, both from ThermoFisher Scientific) for 2 h at RT. Membranes were then washed, revealed using enhanced chemiluminescence substrate (ECL; GE Healthcare) and visualised with an imaging system (Chemidoc, RRID:SCR_019037). Then, membranes were re-probed for anti-α-tubulin antibody (1:20,000, Sigma-Aldrich Cat# T6074, RRID:AB_477582) for protein load control. Densitometric analysis of protein bands was performed using Image Lab Software (BioRad, RRID: SCR_014210). The relative densities of Cx43 and phospho-Cx43 were normalised to α-tubulin and expressed as percentage of the respective control condition.

### Astrocytic cultures

Primary cultures of astrocytes were prepared following a previously used protocol [20]. Briefly, mixed glial cultures were obtained from the cerebral cortex of 2–5 days Wistar rats, grown in astrocyte medium [DMEM, supplemented with 10% foetal bovine serum, penicillin (100 U/mL), streptomycin (100 μg/ mL), HEPES (6 g/L), and sodium bicarbonate (0.84 g/L)] and maintained at 37 °C in a humified 5% CO_2_ incubator for 10–15 days until reaching confluency. Microglia cells were removed by mechanical shaking of the mixed cultures in an orbital shaker for 4 h at 200 rpm and astrocytes detached by a mild trypsinization procedure, re-seeded at a density of 5 × 10^4^ cells/coverslip, and maintained in culture for 2–3 days prior to EtBr uptake.

Based on our previous experience [42], we manipulated different transducing systems to explore the mechanisms involved in the regulation of astrocytic hemichannels, namely using the cAMP analogue and activator of protein kinase A (PKA) 8-Br-cAMP (8-bromoadenosine-3',5'-cyclic monophosphate, 5 µM; Tocris), the protein kinase C (PKC) inhibitor GF109203X (2-[1-(3-dimethylaminopropyl)indol-3-yl]-3-(indol-3-yl)maleimide, 5 µM; Tocris), and the PKC activator PMA (phorbol 12-myristate 13-acetate, Ascent Scientific, 10 ng/mL). The concentrations of the compounds used were supramaximal, but selective for their targets (see [42]). All drugs were applied to cultured astrocytes 30 min prior to challenge with Aβ_1-42_ (1 μM, 24 h), a concentration higher than that used in slices but that we previously defined to be required to trigger astrocyte reactivity and dysfunction in cells obtained from newborn rodents [20–22].

Following the exposure to these pharmacological agents, the activity of hemichannels was assessed through EtBr uptake as previously described [20]. Briefly, astrocytes were exposed for 10 min to EtBr (5 μM) in HBSS solution (137 mM NaCl, 5.4 mM KCl, 0.34 mM Na_2_HPO_4_, 0.44 mM KH_2_PO_4_, 2.7 mM glucose, 1.2 mM CaCl_2_, pH 7.4), washed, fixed with 4% PFA solution and the nuclei stained with Hoechst 33,258. Following three rinses, coverslips were mounted with Fluoromount™ aqueous mounting medium. Images were taken from five random fields under epifluorescence microscopy (Zeiss, Axio Imager Z2 microscope, RRID:SCR_018856 with AxioVision Imaging System, RRID:SCR_002677 version 4.8) and analysed by FIJI-ImageJ software (RRID:SCR_002285).

### Statistical analysis

Data are presented as mean ± SEM of the indicated number (*n*) of animals (in the case of behaviour and Western blot analysis) or hippocampal slices from different animals (for EtBr uptake quantification). In the case of data from tri-dimensional reconstructions of astrocytic processes, *n* refers to the number of cells reconstructed. Comparisons between experimental groups were performed with two-way ANOVA followed by Tukey's or Sidak's multiple comparisons test. Comparison between two experimental conditions was performed using either a paired or unpaired Student's *t* test. To identify differences between strategies used in the Morris water maze test, a table of contingency was built and a Fisher’s exact test of independence was performed. Differences in astrocytic morphometric analysis between WT and APP/PS1 mice (Sholl analysis) were assessed by multiple Student's *t* test using the Holm–Sidak method for correction of multiple comparisons. Statistical significance was set for *p* values < 0.05. All statistical tests were carried out using Graphpad Prism software (version 8.0.1, RRID:SCR_002798).

## Results

### APP/PS1 mice exhibiting memory deficits present morphological alterations of hippocampal astrocytes

**Fig. 1:**
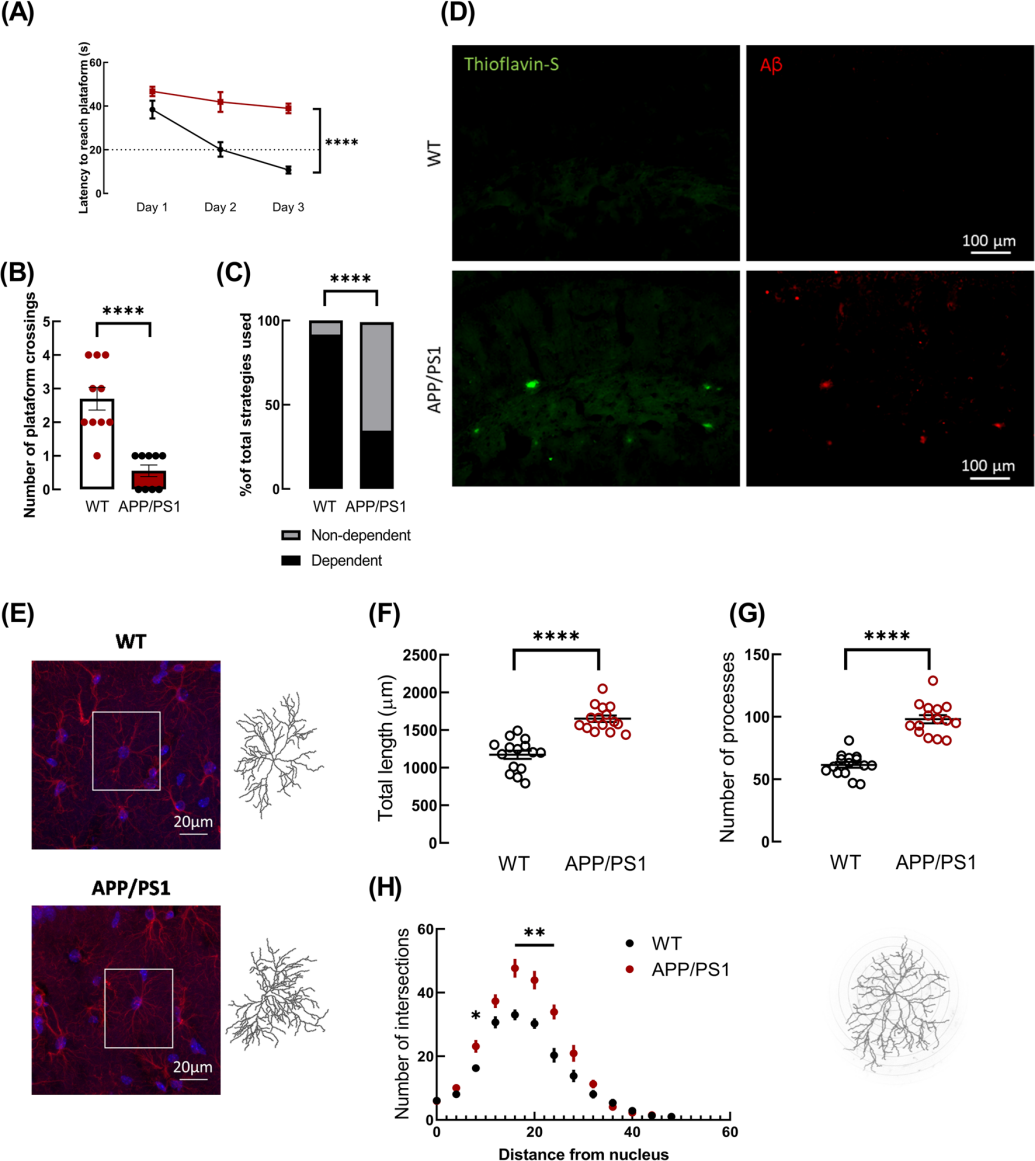
APP/PS1 mice displayed deficits in hippocampal-dependent memory, an accumulation of Aβ and of Aβ plaques and an increased astrocytic arbor complexity in the hippocampus. **A** In the Morris water maze test, APP/PS1 mice displayed a higher latency to find the hidden platform location in the acquisition learning curve than WT mice. ****p < 0.0001, two-way ANOVA followed by a post hoc Sidak’s multiple comparisons test. **B** APP/PS1 mice showed an impairment in spatial memory since the number of crossings of the platform location was significantly lower than WT mice in probe trial of the Morris water maze test. ****p < 0.0001, unpaired Student’s *t* test. **C** Analysis of the search strategy patterns unveiled that WT mice preferentially used strategies implicating the hippocampus, whereas APP/PS1 mice mostly used strategies not dependent on the hippocampus to discover the hidden platform location in the probe trial of the Morris water maze. ****p < 0.0001, Fisher’s exact test of independence. Data are presented as mean ± SEM of *n* = 9–10 mice. **D** Representative images of thioflavin-S staining (left column, green) and Aβ immunolabelling (right column, red) disclosed the presence of amyloid-β plaques in the hippocampus of APP/PS1 but not of WT mice. Scale bar: 100 μm. **E** Representative images of GFAP immunolabelling (red) using a magnification of 63 × in slices from WT and APP/PS1 mice showing the morphology of hippocampal astrocytes. Nuclei were stained with DAPI (blue). Scale bar: 20 μm. Representative fillings of tri-dimensionally reconstructed astrocytes are shown for each group: APP/PS1 mice exhibited an increase in morphological complexity by evaluating **F** total length, **G** number of processes, and **H** Sholl analysis as compared with astrocytes from WT mice. Data are mean ± SEM of 15 astrocytes (from three different mice). ****p < 0.0001, unpaired Student's *t* test and *p < 0.05, **p < 0.01 in Sholl analysis through multiple *t* tests

Learning and memory assessment using the Morris water maze test showed that WT mice displayed a normal learning pattern as they improved their performance throughout the acquisition stage reaching the escape latency criterium (20 s) on day 3 (day 1: 38.43 ± 4.07 s vs. day 3: 10.73 ± 1.57 s, *n* = 10); in contrast, APP/PS1 mice did not reach the escape latency criterium (day 1: 46.72 ± 2.12 s vs. day 3: 38.94 ± 2.20 s, *n* = 9). Two-way ANOVA identified the genotype as a source of variation (*F*_1,17_ = 56.63, *p* < 0.0001), and post hoc Sidak’s multiple comparisons test showed a statistically significant difference between WT and APP/PS1 mice on day 2 (20.15 ± 3.30 s vs. 41.90 ± 4.55 s, *p* < 0.0001) and on day 3 (10.73 ± 1.57 s vs. 38.94 ± 2.20 s, *p* < 0.0001, Fig. 1A). In the probe trial, 24 h following the acquisition stage, APP/PS1 mice crossed the platform location significantly fewer times than WT mice (WT: 2.70 ± 0.34, *n* = 10, vs. APP/PS1: 0.56 ± 0.18, *n* = 9, *p* < 0.0001, *t*_17_ = 5.482, Fig. 1B). Thus, APP/PS1 mice displayed impaired hippocampal-dependent memory. When analysing the search patterns, we observed that APP/PS1 preferentially resorted to non-hippocampal-dependent strategies (only 33% of APP/PS1 mice used strategies dependent on the hippocampus) whereas 90% of WT mice used hippocampal-dependent strategies to find the location of the hidden platform (*p* < 0.0001, Fig. 1C).

Next, we checked for the presence of Aβ accumulation and deposition in the hippocampus of APP/PS1 mice. Figure 1D illustrates the accumulation of Aβ in the hippocampus of APP/PS1 mice, and thioflavin-S staining further supports the existence of an accumulation of these peptides, suggesting the presence of amyloid plaques. By contrast, in the hippocampus of WT mice no thioflavin-S staining was observed. Additionally, we investigated whether APP/PS1 mice displayed alterations in astrocytic arbor complexity through tri-dimensional reconstructions of astrocytes in the CA1 hippocampal region. Astrocytes of APP/PS1 mice displayed a significant increase in the total length of processes (APP/PS1: 1651.15 ± 43.18 µm vs WT: 1171.43 ± 54.27 µm, *n* = 15 astrocytes from three mice, *p* < 0.0001, *t*_28_ = 6.917, Fig. 1F) and also in the number of processes (APP/PS1: 98.07 ± 3.30 vs WT: 61.47 ± 2.30, *n* = 15 astrocytes from three mice, *p* < 0.0001, *t*_28_ = 9.098, Fig. 1G) relatively to hippocampal astrocytes from WT mice. This suggests an increment in morphologic complexity of hippocampal astrocytes in APP/PS1 compared to WT mice. These results were supported by Sholl analysis which unveiled a significant augmentation of the astrocytic arbor complexity of APP/PS1 compared to WT mice at 8 µm and at 16–24 µm from the central soma (8 µm: *p* = 0.0312, 16 µm: *p* = 0.0023, 20 µm: *p* = 0.0035 and 24 µm: *p* = 0.0033, Fig. 1H).

### Astrocytic Cx43 hemichannel activity is increased in the hippocampus of APP/PS1 mice

**Fig. 2:**
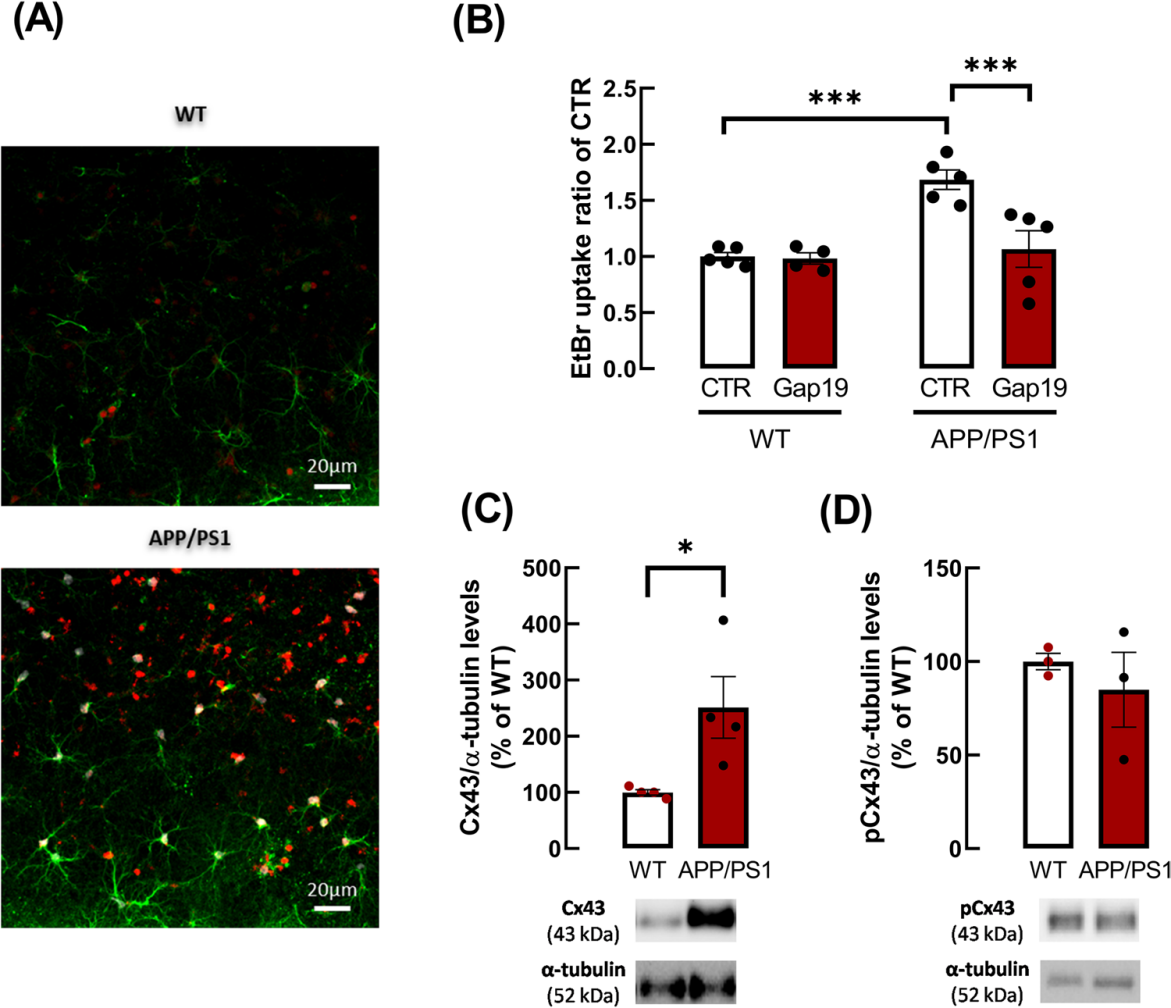
APP/PS1 mice showed an increase of Cx43 hemichannel activity in hippocampal astrocytes and an increase of Cx43 levels. **A** Representative images of EtBr fluorescence signal (red) in astrocytes in hippocampal slices of WT and of APP/PS1 mice. Astrocytes were immuno-labelled with anti-GFAP (green) and nuclei were stained with Hoechst 33,258 (blue). Scale bars: 20 μm. **B** EtBr uptake was significantly higher in hippocampal astrocytes of APP/PS1 mice than in WT mice. The selective blockade of Cx43 hemichannels significantly reduced EtBr uptake in astrocytes of APP/PS1 mice, indicating the involvement of these channels in EtBr uptake. Hippocampal slices from APP/PS1 mice were treated with the Cx43 hemichannel inhibitor, Gap19 (250 µM, 90 min), prior to EtBr uptake assay. ***p < 0.001, two-way ANOVA followed by post hoc Sidak’s multiple comparisons test. The activity of hemichannels was assessed through the fluorescence intensity of EtBr signal, per astrocyte (GFAP^+^cells) nucleus volume (µm^3^) from WT or APP/PS1 mice and expressed as a ratio of control condition. Data are mean ± SEM of five independent experiments. **C** Levels of total Cx43, but not of phospho-Cx43 at Ser 368 (**D**), were increased in gliosomes (membranes from astrocytic processes) obtained from the hippocampus of APP/PS1 relatively to WT mice. The ratio between immunoreactivities for total Cx43 or for phospho-Cx43 at Ser 368 to α-tubulin levels were expressed as a percentage of values obtained in WT mice. Data are mean ± SEM of 3–4 independent experiments. *p < 0.05, unpaired Student’s *t* test. Representative immunoblots for Cx43, phospho-Cx43 and α-tubulin are shown below the average bar graphs

The activity of astrocytic hemichannels was assessed in hippocampal slices using the ethidium bromide (EtBr) uptake assay. Data showed a significant effect of the genotype in EtBr uptake by hippocampal astrocytes (*p* = 0.0179, *F*_1,8_ = 8.809) and further analysis with a post hoc multiple comparisons test unveiled a significant increase of EtBr uptake in hippocampal astrocytes of APP/PS1 compared with WT mice (1.00 ± 0.036 for WT vs 1.69 ± 0.087 for APP/PS1, *n* = 5, *p* = 0.0004, Fig. 2B). To investigate the contribution of Cx43 to the altered hemichannel activity in APP/PS1 mice, Cx43 hemichannels were selectively blocked with Gap19 [43]. Two-way ANOVA showed a significant effect of Gap19 (*p* = 0.0014, *F*_1,7_ = 26.13) and an interaction between Gap19 and genotype (*p* = 0.0015, *F*_1,7_ = 25.35). Sidak’s multiple comparisons tests disclosed a significant effect of Gap19 on EtBr uptake by hippocampal astrocytes of APP/PS1 mice (1.685 ± 0.087 for CTR vs 1.066 ± 0.163 for Gap19, *n* = 5, *p* = 0.0003). Thus, Cx43 hemichannels were major contributors to the EtBr uptake and their blockade rescued the dysfunctional hemichannel activity, restoring astrocytic EtBr uptake to levels similar to WT (Fig. 2B).

Given the relevant role of Cx43 hemichannels in the altered EtBr uptake in APP/PS1 mice, we further evaluated putative alterations of Cx43 levels in gliosomes (membranes of astrocytic processes) obtained from the hippocampus of APP/PS1 compared to WT mice. Data showed a substantial enhancement of Cx43 levels in gliosomes from APP/PS1 mice when compared with WT mice (100.00 ± 4.68% for WT vs. 251.33 ± 55.02% for APP/PS1 mice, *n* = 4, *p* = 0.0337, *t*_6_ = 2.741, Fig. 2D). Since the activity of hemichannels is modulated by phosphorylation, we also evaluated alterations of Cx43 phosphorylation at Ser368 and no alterations were observed between gliosomes of WT and APP/PS1 mice (100.00 ± 43.85% for WT vs. 87.95 ± 32.30% for APP/PS1, *n* = 4, *p* = 0.8323, *t*_6_ = 0.2212, Fig. 2E). The ratio between pCx43 and total Cx43 was 1.00 ± 0.12 for WT mice and of 0.64 ± 0.12 for APP/PS1 mice, supporting an increase in total Cx43 levels.

### Hemichannel activity is affected in hippocampal astrocytes at early AD stages

**Fig. 3:**
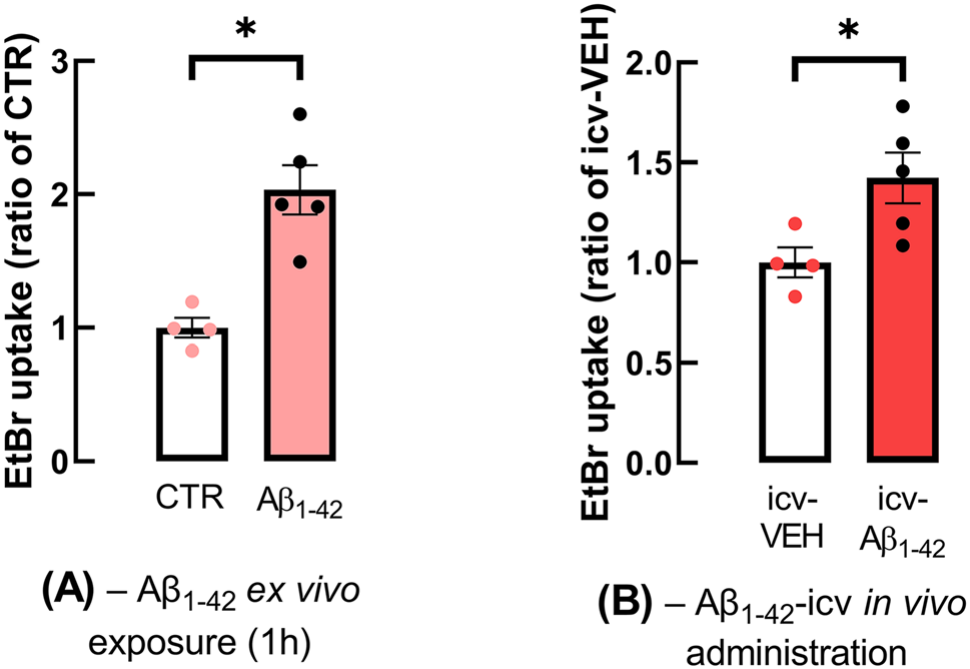
Hemichannel activity was also enhanced in astrocytes of models mimicking early AD. The activity of hemichannels was augmented in astrocytes of hippocampal slices obtained from **A** WT mice and incubated with Aβ_1-42_ (50 nM, 60 min) and **B** from WT mice administrated icv with Aβ_1-42_ (icv-Aβ_1-42_), compared to control (or icv-vehicle administration) WT mice, being the EtBr uptake assay performed after 15 days of Aβ_1-42_- or vehicle-icv administration. The activity of hemichannels was evaluated by the mean fluorescence intensity of EtBr signal per astrocyte nucleus volume and expressed as a ratio of control for each experimental condition. Data are mean ± SEM of 4–5 independent experiments. *p < 0.05, paired for (**A**) or unpaired for (**B**) Student's *t* test

The aforementioned data showed that adult APP/PS1 mice displayed hippocampal-dependent memory deficits at 9 months of age, exhibiting also Aβ plaques in parallel with altered astrocytic morphology, heighten hemichannel activity and increased Cx43 levels in the hippocampus. However, several evidences suggest that soluble Aβ oligomer rather than Aβ deposits primarily contribute to neuronal dysfunction, synaptic loss and early memory deficits (reviewed in [1, 44]) as well as with astrocytic dysfunction in early AD stages [34, 35]. Therefore, we next evaluated hemichannel activity in conditions mimicking early AD, using hippocampal slices directly superfused with Aβ_1-42_ (50 nM, 60 min) and hippocampal slices collected from adult mice intracerebroventricularly injected with Aβ_1-42_ (icv-Aβ_1-42_), which we previously showed to cause hippocampal synaptic plasticity and memory impairment after 15 days of peptide administration without evidence of Aβ deposits [24, 34]. In hippocampal slices exposed to Aβ_1-42_, the EtBr uptake by astrocytes was significantly higher than that of control slices (2.03 ± 0.185, *n* = 5, for Aβ_1-42_ vs. 1.00 ± 0.075, *n* = 4, for CTR, *p* = 0.0475, t_3_ = 3.250, Fig. 3A). Similarly, the icv Aβ_1-42_ administration also resulted in an enhancement of EtBr uptake by hippocampal astrocytes compared to vehicle-injected control mice (icv-VEH: 1.00 ± 0.075, *n* = 4 vs. icv-Aβ_1–42_: 1.42 ± 0.127, *n* = 5, *p* = 0.0323, t_7_ = 2.664, Fig. 3B).

### icv-Aβ_1-42_ peptides administration affects Cx43 phosphorylation at Ser368

**Fig. 4:**
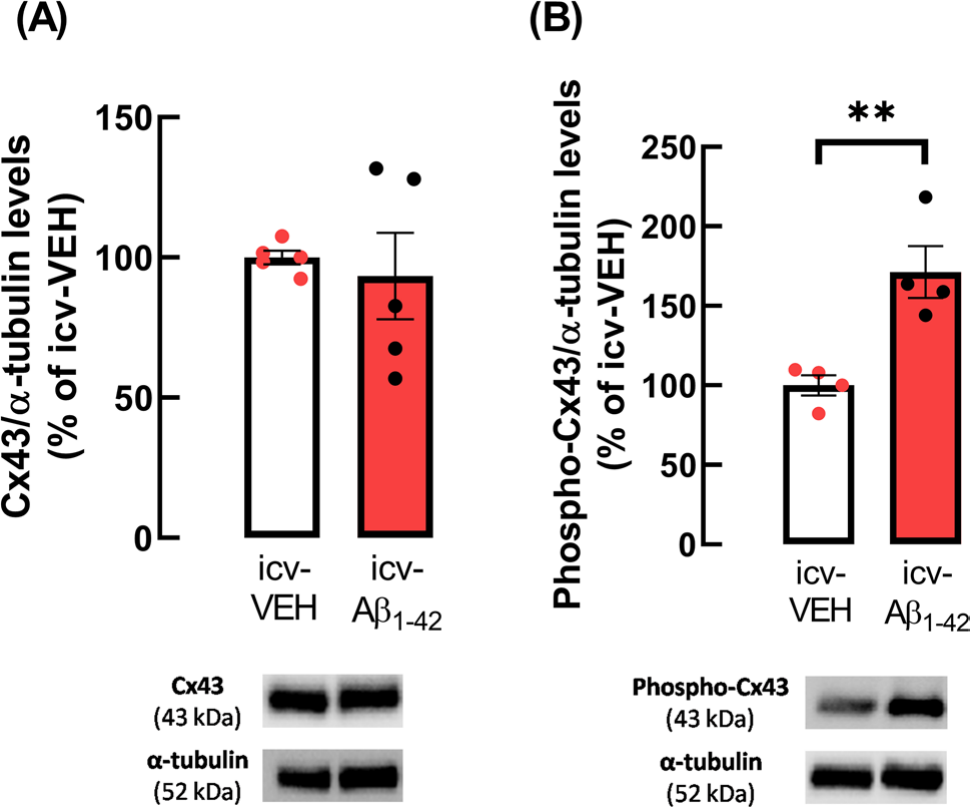
Aβ_1-42_-icv administration increased Cx43 phosphorylation at Ser368 in hippocampal gliosomes of mice mimicking early AD stages. Although no alterations were observed in **A** Cx43 levels, an enhancement in **B** phospho-Cx43 levels (at Ser368) was observed in gliosomes obtained from the hippocampus of mice icv administrated with Aβ_1-42_ in comparison with icv-vehicle (VEH)-injected control mice. Data are ratios between Cx43 or phospho-Cx43 and α-tubulin (loading control protein) immunoreactivities expressed as percentage of the control condition (icv-VEH). Data are mean ± SEM of 4–5 independent experiments. **p < 0.01, unpaired Student’s *t* test. Representative immunoblots for Cx43, phospho-Cx43 and α-tubulin are shown

Since in APP/PS1 mice, the enhancement of the activity of astrocytic hemichannels was linked with an increase of Cx43 levels, we next analysed if Aβ_1-42_-icv administration affected Cx43 levels in hippocampal gliosomes. Curiously, although no alterations were found in Cx43 levels (WT: 100.00 ± 2.41% vs. Aβ: 93.29 ± 15.45%, *n* = 5, *p* = 0.6836, *t*_8_ = 0.4227, Fig. 4A), a significant increase in the levels of Cx43 phosphorylated at Ser368 residue was observed in gliosomes from the hippocampus of Aβ_1-42_-icv injected compared to vehicle-injected control mice (WT: 100.00 ± 6.30% vs. Aβ: 171.28 ± 16.27%, *n* = 4, *p* = 0.0065, *t*_6_ = 4.085, Fig. 4B). Accordingly, the ratio phospho-Cx43(Ser368)/total Cx43 was 0.98 ± 0.05 for icv-VEH mice and 1.56 ± 0.05 for icv-Aβ_1-42_ mice.

### A_2A_R modulate the activity of hemichannel activity in hippocampal astrocytes

**Fig. 5:**
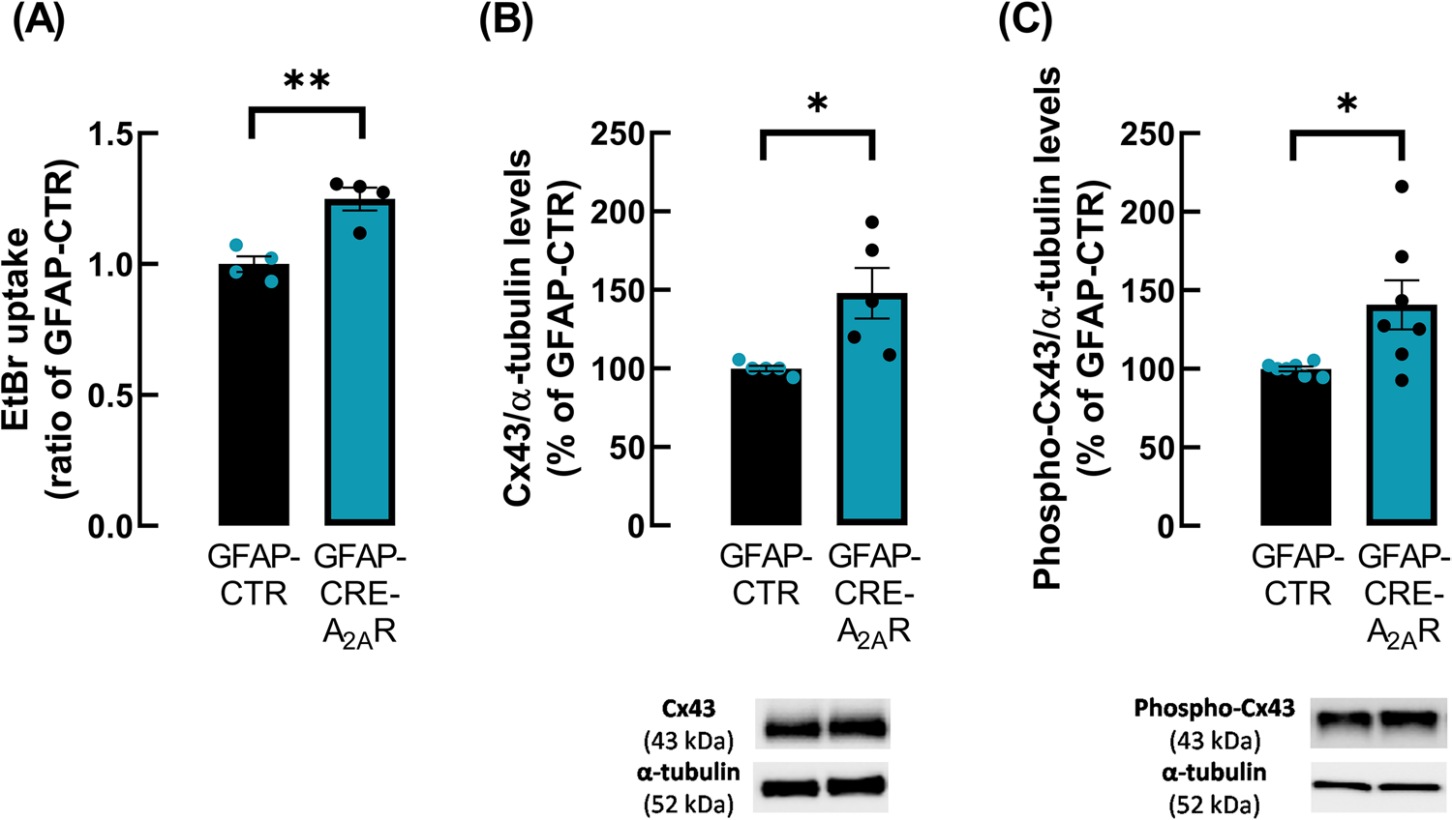
Astrocytic A_2A_R genetic silencing increased the activity of hemichannels and the levels of Cx43 and of phospho-Cx43 at Ser368 in hippocampal astrocytes. **A** Deletion of A_2A_R selectively in astrocytes led to the enhancement of EtBr uptake in hippocampal astrocytes. Hemichannel activity was evaluated by the mean fluorescence intensity of EtBr signal per astrocyte nucleus volume expressed as a ratio of control condition (GFAP-CTR) in hippocampal slices from GFAP-CTR and GFAP-CRE-A_2A_R mice. The levels of **B** Cx43 and of **C** phospho-Cx43 at Ser368 were increased in gliosomes obtained from the hippocampus of GFAP-CRE-A_2A_R compared to GFAP-CTR mice. Immunoreactivity ratio of Cx43 and of phospho-Cx43 to α-tubulin were expressed as percentage of GFAP-CTR. Representative immunoblots for Cx43, phospho-Cx43 and α-tubulin are shown. Data are mean ± SEM of 4–7 independent experiments. *p < 0.05, **p < 0.01 unpaired Student’s *t* test

Next, we investigated whether astrocytic A_2A_R were involved in the modulation of the activity of hemichannels in astrocytes of hippocampal slices. For this purpose, we used a mouse model with astrocytic A_2A_R genetically silenced in the hippocampus, as described above (“Animals and surgeries" section). Data showed that silencing astrocytic A_2A_R increased EtBr uptake by astrocytes when compared with the control mice (1.25 ± 0.044 for GFAP-CRE-A_2A_R vs. 1.00 ± 0.031 for GFAP-CTR, *n* = 4, *p* = 0.0035, *t*_6_ = 4.641, Fig. 5A). Additionally, we investigated if silencing astrocytic A_2A_R affected the total and phosphorylated levels of Cx43. Data showed a significant increase in the levels of Cx43 (GFAP-CRE-A_2A_R: 147.97 ± 16.06% vs. GFAP-CTR: 100.00 ± 1.78% *n* = 5, *p* = 0.0179, *t*_8_ = 2.968, Fig. 5B) and of phospho-Cx43 at Ser368 (GFAP-CRE-A_2A_R: 140.78 ± 15.70% vs. GFAP-CTR: 100.00 ± 1.49% *n* = 7, *p* = 0.0238, *t*_12_ = 2.586, Fig. 5C), being the ratio phosphoCx43/Cx43 similar for both groups (GFAP-CTR: 1.00 ± 0.09 vs. GFAP-CRE-A_2A_R: 0.098 ± 0.12). These data confirm that A_2A_R control the activity of hemichannels composed by Cx43 in hippocampal astrocytes, increasing Cx43 levels and Cx43 phosphorylation.

### A_2A_R regulate alterations in astrocytic hemichannel activity triggered by an acute challenge with Aβ_1-42_

**Fig. 6:**
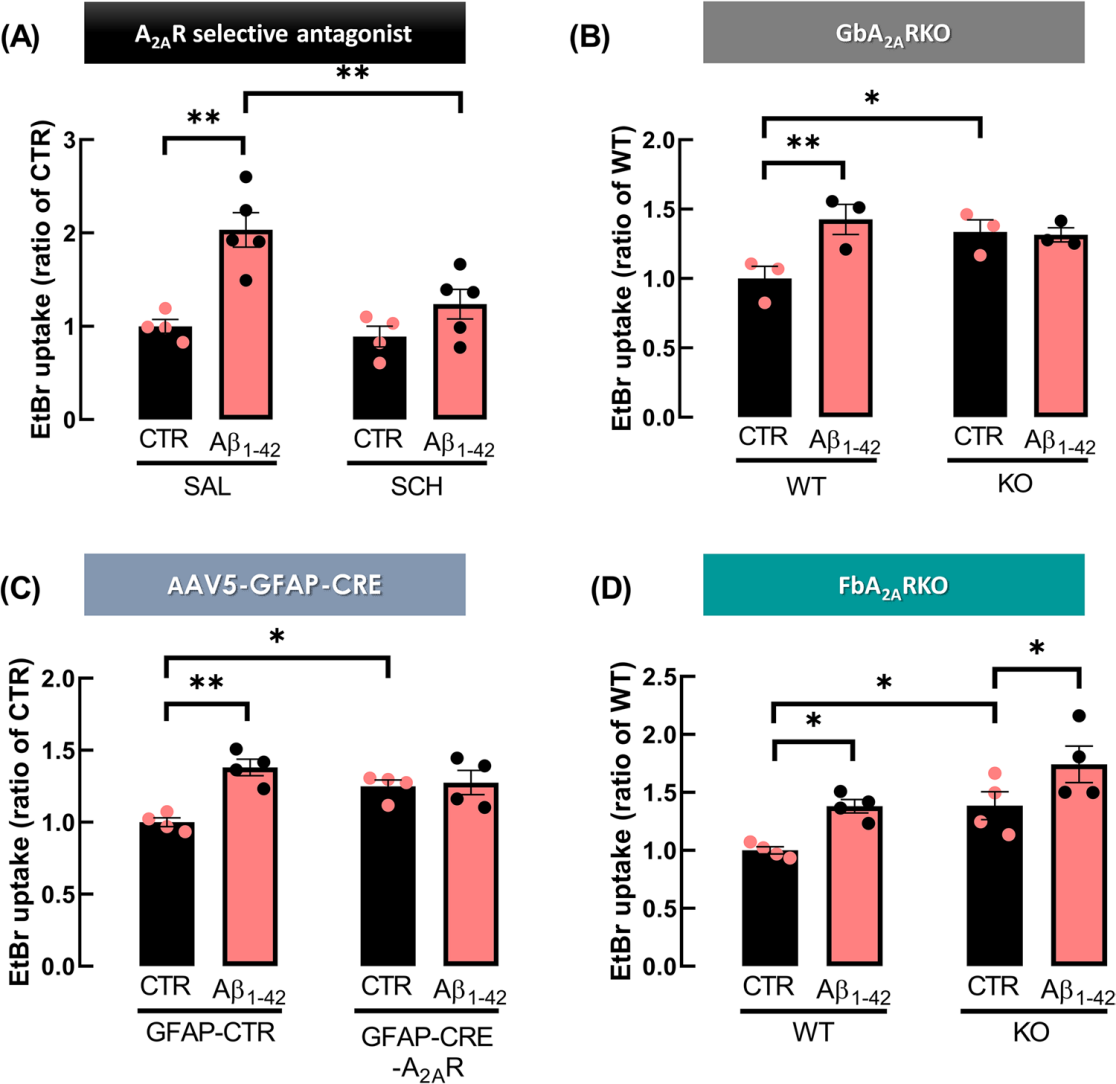
A_2A_R regulated alterations in hemichannel activity caused by an acute challenge with Aβ_1-42_. **A** The enhancement in astrocytic hemichannel activity driven by the acute exposure of hippocampal slices to Aβ_1-42_ was prevented by A_2A_R selective blockade. Hippocampal slices were pre-incubated with SCH58261 (SCH, 50 nM) for 30 min prior to challenge with Aβ_1-42_ (50 nM, 60 min), before the EtBr uptake assay. **B** In GbA_2A_RKO and **C** GFAP-CRE-A_2A_R mice, Aβ_1-42_ failed to increase EtBr uptake in hippocampal astrocytes, whereas **D** in FbA_2A_RKO mice, Aβ_1-42_ augmented astrocytic EtBr uptake. The activity of hemichannels was assessed through the fluorescence intensity of EtBr signal, per astrocyte (GFAP^+^cells) nucleus volume, expressed as the mean of each animal (*n*) and normalised to the control condition. Data are mean ± SEM of 3–5 independent experiments, corresponding to different mice *p < 0.05, **p < 0.01, two-way ANOVA post hoc Sidak’s multiple comparisons test. Please note that the control values in panel A is the same as in Fig. 3A and in panel C is the same as Fig. 5A

To further explore the role of A_2A_R in the dysregulation of astrocytic hemichannel activity triggered by Aβ_1-42_ peptides, we resorted to different approaches. First, a pharmacological strategy was employed where hippocampal slices were treated ex vivo with A_2A_R selective antagonist, SCH58261, prior to Aβ_1-42_ challenge. Two-way ANOVA revealed an interaction between Aβ_1-42_ and SCH58261 (*F*_1,14_ = 5.241, *p* = 0.0381), and post hoc analysis with Tukey’s multiple comparisons test revealed that A_2A_R selective blockade prevented the increase in EtBr uptake triggered by Aβ_1-42_ (Aβ_1-42_: 2.03 ± 0.185, *n* = 5, vs. SCH58261 + Aβ_1-42_: 1.24 ± 0.158, *n* = 5, *p* = 0.0066, Fig. 6A, of noting that the Aβ_1-42_/SAL data are the same presented in Fig. 3A). The administration of SCH58261 per se did not significantly affect EtBr uptake, as compared with control conditions (SCH: 0.891 ± 0.112 vs CTR SAL: 1.000 ± 0.075; *p* = 0.9604, Fig. 6A). Moreover, we further resorted to A_2A_R genetic silencing. In GbA_2A_RKO mice, two-way ANOVA showed an interaction between genotype and Aβ_1-42_ (*p* = 0.0035, *F*_1.4_ = 38.24) and Sidak’s multiple comparisons tests unveiled that the amount of EtBr taken by astrocytes in hippocampal slices from GbA_2A_RKO mice was significantly higher than in WT mice (KO: 1.34 ± 0.087 vs. WT: 1.00 ± 0.088, *n* = 3, *p* = 0.0493). Furthermore, Aβ_1-42_ superfusion of hippocampal slices enhanced EtBr uptake in WT mice (Aβ_1-42_: 1.42 ± 0.109 vs. CTR: 1.00 ± 0.088, *n* = 3, *p* = 0.0023), but did not affect EtBr uptake by astrocytes in hippocampal slices from GbA_2A_RKO mice (CTR: 1.34 ± 0.087 vs. Aβ_1-42_: 1.32 ± 0.051, *n* = 3, *p* = 0.9069, Fig. 6B). Interestingly, despite no significant differences were detected in Cx43 levels (WT: 108.65 ± 10.92% vs. KO: 106.26 ± 9.88%, *n* = 6, *p* = 0.8746, *t*_10_ = 0.1619, supplementary data Figure S2A), a downregulation in Cx43 phosphorylation at Ser368 was observed in gliosomes obtained from the hippocampus of GbA_2A_RKO mice (WT: 100.00 ± 9.74 vs. KO: 67.24 ± 3.57, *n* = 4, *p* = 0.0196, *t*_6_ = 3.158, supplementary data Figure S2B). When A_2A_R genetic deletion is selectively carried out in astrocytes, two-way ANOVA showed an interaction between Aβ_1-42_ and the effective viral construct administrated (*p* = 0.0183, *F*_1,6_ = 10.34) and Sidak’s multiple comparisons test showed a lack of effect of Aβ_1-42_ superfusion in EtBr uptake by astrocytes in hippocampal slices of GFAP-CRE-A_2A_R mice (CTR: 1.25 ± 0.040 vs. Aβ_1-42_: 1.28 ± 0.084, *n* = 4, *p* = 0.9320), whereas in GFAP-CTR mice Aβ_1-42_ significantly increased the amount of EtBr taken up by astrocytes (Aβ_1-42_: 1.38 ± 0.058, *n* = 4 vs. CTR: 1.00 ± 0.031, *n* = 4, *p* = 0.0054, Fig. 6C, noting that CTR data of GFAP-CRE-A_2A_R and of GFAP-CTR mice are the same data present in Fig. 5A). In contrast, in FbA_2A_RKO mice, two-way ANOVA did not reveal an interaction between genotype and Aβ_1-42_ treatment (*p* = 0.8714, *F*_1,6_ = 0.0286); notwithstanding, it is pertinent to emphasise that a significant effect was observed in the genotype (*p* = 0.0276, *F*_1,6_ = 8.364) and Aβ_1-42_ treatment (*p* = 0.0020, *F*_1,6_ = 27.24). Additionally, Sidak’s multiple comparisons test revealed a significant enhancement in EtBr uptake by hippocampal astrocytes triggered by Aβ_1-42_ in both WT mice (CTR: 1.00 ± 0.031 vs. Aβ_1-42_: 1.38 ± 0.058, *n* = 4, *p* = 0.0177) and FbA_2A_RKO mice (CTR: 1.39 ± 0.120 vs. Aβ_1-42_: 1.74 ± 0.157, *n* = 4, *p* = 0.0234). Curiously, a significant increase in EtBr uptake was also observed in hippocampal astrocytes of FbA_2A_RKO mice when compared with WT mice (KO: 1.39 ± 0.120 vs. WT: 1.00 ± 0.031, *n* = 4, *p* = 0.0443, Fig. 6D), but the Aβ_1-42_ superfusion similarly increased (*p* > 0.05) EtBr uptake in WT (0.38 ± 0.03) and FbA_2A_RKO (0.35 ± 0.04) mice (Fig. 6D). Surprisingly, the analysis of gliosomal preparations from the hippocampus of FbA_2A_RKO mice revealed neither alterations of Cx43 levels (WT: 100.00 ± 2.47% vs. KO: 102.35 ± 7.58%, *n* = 5, *p* = 0.7760, *t*_8_ = 0.2943, supplementary data Figure S2C) nor of Cx43 phosphorylation at Ser368 (WT: 100.00 ± 8.53% vs. KO: 81.81 ± 16.02%, *n* = 6, *p* = 0.3397, *t*_10_ = 1.003, supplementary data Figure S2D). Taken together, these data identified the importance of astrocytic A_2A_R in the modulation of hemichannel activity of hippocampal astrocytes in pathological conditions mimicking early AD stages.

### The dysfunctional hemichannel activity triggered by Aβ seems to involve a PKC-mediated signalling pathway

**Fig. 7:**
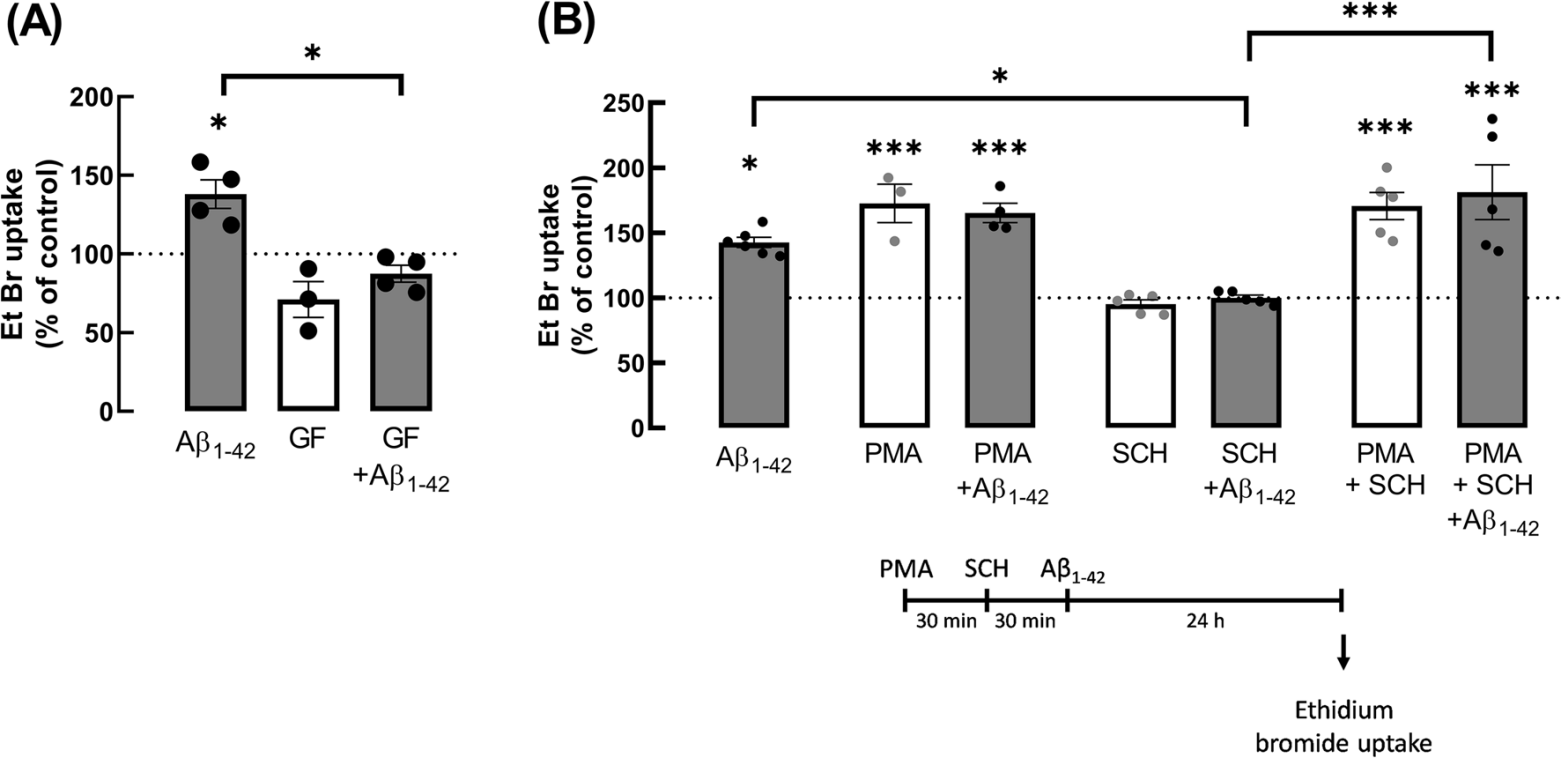
The increased hemichannel activity in cultured astrocytes exposed to Aβ_1-42_ was regulated by PKC. **A** PKC inhibition with GF109203X (GF) prevented the effect of Aβ_1-42_ on EtBr uptake in astrocytes. Cultured astrocytes were exposed to GF (5 µM) for 30 min prior to challenge with Aβ_1-42_ (1 µM, 24 h). Data are mean ± SEM of 3–4 independent experiments. *p < 0.05, two-way ANOVA post hoc Tukey’s multiple comparisons test. **B** On the other hand, PKC activation with PMA (phorbol 12-myristate 13-acetate, 10 ng/mL) mimicked the effect of Aβ_1-42_ on EtBr uptake in astrocytes. In the presence of PMA, the selective A_2A_R antagonist SCH58261 (SCH) did not blunt the Aβ_1-42_-induced increase in EtBr uptake by astrocytes. The selective A_2A_R antagonist SCH58261 (SCH) was no longer able to blunt the increase in EtBr uptake in astrocytes pre-treated with PMA. Cultured astrocytes were exposed to PMA (10 ng/mL, 30 min), then SCH was added (50 nM, 30 min) and finally astrocytes were exposed to Aβ_1-42_ (1 µM, 24 h) in the presence of the previously added compounds, as depicted in the timeline shown. The activity of hemichannels was assessed through the mean fluorescence intensity of retained EtBr in the nucleus upon the subtraction of background values. Data are presented as percentage values relative to non-treated control cells (100%) and are mean ± SEM of 3–5 independent experiments. *p < 0.05, ***p < 0.001, three-way ANOVA post hoc Tukey’s multiple comparisons test

Although A_2A_R are pleiotropic receptors, they are mainly recognised to engage the PKA-mediated transducing system (reviewed in [46]). However, since we previously reported that the Aβ_1-42_-induced increase of hemichannel activity is paralleled by increased Cx43 phosphorylation in residue Ser368, which is phosphorylated by PKC [47, 48], and this effect was mimicked by A_2A_R activation and prevented by selective A_2A_R blockade [20], we next characterised the transducing system involved in the control of hemichannel activity in cultured hippocampal astrocytes. We observed that PKA activation with the cAMP analogue 8-Br-cAMP (5 µM) did not affect EtBr uptake (101.16 ± 9.01% vs. CTR: 100%, *t*_3_ = 0.1284, *p* = 0.9060), whereas the activation of PKC with a phorbol ester analogue (PMA, phorbol 12-myristate 13-acetate, 10 ng/mL) increased hemichannel activity (128.10 ± 4.52% vs. CTR: 100%, *t*_3_ = 6.222, *p* = 0.0084, supplementary data Figure S3A). Moreover, PMA significantly enhanced Cx43 phosphorylation at Ser368 (135.1 ± 10.82% vs. CTR: 99.00 ± 6.74%, *t*_6_ = 2.829, *p* = 0.0300, supplementary data Figure S3B). These data suggest that the enhancement of hemichannel activity triggered by Aβ_1-42_ might involve the recruitment of a PKC-mediated pathway. In support of this hypothesis, PKC inhibition with GF109203X (5 µM) prevented the enhancement of hemichannel activity caused by Aβ_1-42_ (Fig. 7B). Thus, two-way ANOVA unveiled a significant effect of GF109203X (*p* = 0.0002, *F*_1,11_ = 30.14) and of Aβ_1-42_ (*p* = 0.0031, *F*_1,11_ = 14.16) in EtBr taken up by cultured astrocytes. Further analysis with Tukey multiple comparisons test showed that GF109203X significantly reduced EtBr uptake in astrocytes exposed to Aβ_1-42_ (87.51 ± 5.36% vs. 138.05 ± 9.14% for Aβ_1-42_, *p* = 0.0016) when compared to astrocytes exposed only to Aβ_1-42_. Interestingly, GF109203X appeared to decrease EtBr uptake by hippocampal astrocytes, but this effect did not reach statistical significance (71.13 ± 11.39%, *p* = 0.0805). Moreover, PKC activation with PMA mimicked the effect of Aβ_1-42_ on EtBr uptake in cultures astrocytes (Fig. 7A). Three-way ANOVA disclosed a significant effect of PMA (*F*_1,31_ = 83.02, *p* < 0.0001) and Tukey multiple comparisons tests revealed a significant increase of EtBr uptake relatively to non-treated control (100%), in astrocytes challenged with either Aβ_1-42_ alone (142.56 ± 3.94%, *p* = 0.0279), PMA alone (172.58 ± 14.77%, *p* = 0.0008) and PMA + Aβ_1-42_ (165.29 ± 7.42%, *p* = 0.0009). Noteworthy, no additivity was observed between the effects of PMA and of Aβ_1-42_ (*p* = 0.9998). To further validate the involvement of a PKC-mediated pathway, PKC was activated followed by A_2A_R blockade prior to Aβ_1-42_ challenge (Fig. 7B). Tukey multiple comparisons tests disclosed that astrocytes exposed to the PKC activator PMA and to the selective A_2A_R antagonist SCH58261 displayed a significant increased EtBr uptake in the absence (170.64 ± 10.46%, *p* = 0.0001) and in the presence of Aβ_1-42_ (181.23 ± 21.07%, *p* < 0.0001) when compared with control astrocytes. Moreover, as can be seen in Fig. 7B, A2AR blockade per se did not affect EtBr uptake (95.18 ± 3.29%, *p* > 0.999) by cultured astrocytes, but prevented alterations triggered by Aβ_1-42_ (99.95 ± 2.22%, *p* = 0.0409), similarly to that previously observed by us [20]. Noteworthy, when astrocytes were exposed to PMA, SCH58261 and Aβ_1-42_, the amount of EtBr taken up by astrocytes was significantly higher than in astrocytes exposed to SCH58261 and Aβ_1-42_ (*p* < 0.0001), but as expected not different (*p* > 0.05) to that observed in astrocytes exposed to PMA and SCH58261 (Fig. 7B). Altogether, the gathered results strongly support the involvement of a signalling pathway mediated by PKC in our experimental conditions, which can be recruited downstream A_2A_R activation.

## Discussion

The present study re-enforces the relevance of astrocytic alterations at the onset of memory deficits and in experimental models of established AD, by showing that the activity of astrocytic hemichannels composed by Cx43 is modified in animal models both of early stages as well as of established AD. Furthermore, the observed different alterations of total Cx43 levels and of phosphorylated Cx43 at Ser368 in different models mimicking different AD stages from in vitro to in vivo suggest that the enhancement in astroglial hemichannel activity might be due to different mechanisms throughout the evolution of AD.

The analysis of astrocyte morphology in the hippocampus of APP/PS1 mice when memory dysfunction became evident, clearly showed global alterations of astrocyte morphology, typified by an increase in arbor complexity and an enhancement in the number and total length of astrocytic processes. This finding is aligned with previous observations that an increase in astrocyte reactivity is linked to the accumulation of Aβ associated with AD, as assessed by the analysis of GFAP immunoreactivity in human samples and animal models of AD [6, 27, 49, 50], namely in APP/PS1 mice [7, 14, 51, 52]. Given the active role of astrocytes in synaptic communication and memory processes [38, 53, 54], these alterations of astrocytes emerge as potential contributors to the deficits in hippocampal-dependent learning and memory observed in the performance of APP/PS1 mice in the Morris water maze test.

Our present study also revealed that the increased reactivity of astrocytes in the AD brain was associated with an increase of astroglial hemichannel activity. In the present study, although we did not use scrambled peptides, we pinpointed the specific contribution of Cx43 to the measured astroglial hemichannel activity by resorting to Gap19, which was previously shown to selectively target Cx43 hemichannels without affecting gap junctions [43]. Accordingly, we observed increased total levels of Cx43 in APP/PS1 mice that display scarce amyloid plaques. This is in agreement with previous studies also reporting an increase of Cx43 protein and mRNA levels in the brain of both patients [49, 55] and animal models [14, 18, 56] with established AD. This increased density of total Cx43 was originally associated with sites of Aβ deposits [14, 49], but was later recognised to be widespread in the afflicted brain regions of AD [18]. It should be mentioned that although we observed an increase in astrocytic process length, number and complexity in APP/PS1 mice, the increase in total Cx43 levels does not seem to be a direct consequence of the increased number of astrocytic processes, because we quantified the levels of total and phosphorylated (at Ser 368) Cx43 in a similar amount of astrocytic processes membranes (gliosomes) of wild-type and APP/PS1 mice.

Interestingly, we now observed that the increased activity of hemichannels in hippocampal astrocytes is an early event of AD pathogenesis, which is maintained in advanced stages of the disease. In early AD modelled by Aβ-icv exposure, there was an increase in Cx43 phosphorylation at Ser368 without alterations of the total levels of Cx43, whereas in hippocampal gliosomes of 9-month-old APP/PS1 mice already displaying sparse Aβ plaques, there was an increase in Cx43 levels without alterations in Cx43 phosphorylation at Ser368. This is in agreement with the ability of soluble forms of Aβ to control Cx43 levels at cell membrane [57], bolstering gliotransmitters release from cultured astrocytes, namely ATP and glutamate [20, 56, 58], which might promote neuronal injury [58]. These findings further indicate that the onset of AD seems to be related with alterations in Cx43 phosphorylation, whereas advanced AD stages involve alterations in Cx43 levels, which is consistent with the progressive increase in Cx43 levels observed in APP/PS1 and 5xFAD mice [59] as well as in AD patients [55]. Although we have not directly explored the impact of increased Cx43-HC activity as a trigger of memory dysfunction, this increased of Cx43 might be a contributing factor for AD-related dysfunction of synaptic plasticity and memory since the genetic silencing of Cx43 affords a neuroprotection against Aβ-induced modifications [55] and improves memory deficits in APP/PS1 mice by increasing synaptic function without affecting amyloid plaque formation or the inflammatory response [18, 59].

We further explored the mechanism involved in the regulation of hemichannel activity by phosphorylation which remains largely unknown in astrocytes. The observed enhancement in Cx43 phosphorylation in Ser368 residue, which is mainly mediated by PKC [60, 61], in hippocampal astrocytes of mice icv administrated with Aβ peptides, is in line with alterations previously reported by us in cultured astrocytes exposed to Aβ peptides [20]. These data suggest an Aβ-induced increase in PKC activity, which was already reported in human astrocytes exposed to Aβ [62] as well as in cortical astrocytes of 5xFAD mice [47]. Thus, we postulate as a working hypothesis that the increased PKC activation and the subsequent enhancement of Cx43 phosphorylation at Ser368 might be responsible for the increased hemichannel activity under AD conditions in hippocampal astrocytes. Interestingly, a recent study showed that an inflammatory stimulus (Thy-1) caused astrocytic Cx43 phosphorylation at Ser373 through a PI3K/AKT signalling pathway, being this phosphorylation related with the increased opening of hemichannels and ATP release [63]. Some studies performed in endothelial and cardiac cells also showed a link between PKC-mediated phosphorylation of Cx43 at Ser368 residue leading to altered hemichannel activity [64] and of gap junctional intercellular communication [48, 64, 65]; however, little is known about the control of Cx43 phosphorylation at Ser368 in astrocytes [66].

A second major advance provided by the present study is the concept that adenosine A_2A_ receptors (A_2A_R) modulate the impact of Aβ in the activity of astrocytic Cx43 hemichannels. Although, we had previously shown that A_2A_R control Cx43 hemichannels in cultured astrocytes exposed to Aβ peptides [20], we now extend our studies to a more complex system, namely mouse hippocampal slices modelling early or advanced stages of AD, in which A_2A_R were genetically silenced in astrocytes and/or in neurons. Our group and others showed that A_2A_R blockade ameliorates synaptic dysfunction and loss in addition to memory deficits in mice icv administered with Aβ_1-42_ [24, 31] and in APP/PS1 mice [25, 26, 67]. This A_2A_R-mediated control of AD-related dysfunction involves an overfunction of neuronal A_2A_R [25, 31, 67], in accordance with the predominant localization of A_2A_R in excitatory synapses in the limbic cortex [68, 69]. However, A_2A_R are also present in astrocytes and the selective manipulation of astrocytic A_2A_R has an impact on memory function [23, 29, 70]. Furthermore, A_2A_R modulate alterations triggered by Aβ in primary cultures of astrocytes, namely decreased glutamate uptake [21], altered Ca^2+^ dynamics [22], and increased hemichannel activity and subsequently ATP release [20], a danger signal in brain disease conditions [71]. We now extend these findings by showing that the Aβ-induced alterations in hemichannel activity in hippocampal slices were abrogated by the pharmacological blockade and genetic silencing of A_2A_R in astrocytes, whereas they were unaffected in hippocampal astrocytes of mice with a selective genetic silencing of A_2A_R in neurons (FbA_2A_RKO); this further re-enforces the conclusion that astrocytic A_2A_R are responsible for the regulation of Aβ-induced alterations in astrocytic hemichannel activity in the hippocampus. Interestingly, A_2A_R also have a relevant role in non-pathological conditions since global, neuronal and astrocytic A_2A_R genetic silencing significantly impacted the activity of astrocytic hemichannels in the hippocampus. Indeed, mice lacking astrocytic A_2A_R displayed an increased hemichannel activity, which is likely a consequence of an increase of total Cx43 and phospho-Cx43 levels (see also [72]). Thus, astrocytic A_2A_R emerge as key modulators of hemichannel activity, which is consistent with the reported physical association between A_2A_R and Cx43 in primary cultures of astrocytes [20]. This provides a molecular rationale to understand that the removal of A_2A_R from the complex with Cx43 can contribute to dysregulate hemichannel activity, which may underlie the observation that the selective silencing of astrocytic A_2A_R results in impairments of synaptic plasticity and deficits in hippocampal-dependent reference memory [23]. Interestingly, we also observed that the genetic silencing of neuronal A_2A_R (using FbA_2A_RKO mice) triggered an enhancement of the activity of hippocampal astrocytic hemichannels relatively to WT littermates, despite no alterations in Cx43 or phospho-Cx43 levels were detected, suggesting that alterations in neuronal function due to A_2A_R deletion can influence astrocytic activity. This idea is in agreement with a study reporting that, under physiological conditions, the activity of Cx43 hemichannels in astrocytes is promoted by neuronal activity, which, in turn, modulates neuronal network function via a purinergic pathway in the olfactory bulb [73]. Altogether our data indicate that astrocytic A_2A_R are crucial to modulate Cx43 hemichannel activity in hippocampal astrocytes under AD-like conditions. Furthermore, our data also provides a tentative rationale for a riddle regarding the impact of astrocytic A_2A_R on memory performance, whereby the genetic elimination of astrocytic A_2A_R is detrimental for memory performance in naïve animals [23, 70] but beneficial in AD mouse models [29]. This is in agreement with the observed increase of hemichannel activity upon deletion of astrocytic A_2A_R in naïve animals and with the lack of additional effects of Aβ on hemichannel activity in hippocampal slices of mice with a genetic deletion of astrocytic A_2A_R.

This opposite role of A_2A_R in naïve animals and in conditions of early AD is likely due to the upregulation of astrocytic A_2A_R [21]. Previous studies have shown that the upregulation of A_2A_R is coupled with an alteration of the transducing signalling system operated by A_2A_R [74]. In fact, whereas A_2A_R are canonically coupled to the activation of adenylate cyclase and generation of cAMP [46], these pleiotropic receptors seem to be mostly coupled to PKC when they are upregulated in disease conditions associated with increased glutamatergic signalling [74], such as in early AD (see e.g. [75–77]). And, in agreement with the impact of PKC on the activity of astrocytic hemichannels, we observed that A_2A_R blockade was not able to prevent Aβ-induced alterations of hemichannel activity when PKC was previously activated, which supports the contention that the recruitment of the PKC pathway is a downstream event to A_2A_R activation under conditions of Aβ exposure. This is in agreement with several previous reports indicating the involvement of PKC in the signalling of glial A_2A_R [74, 78, 79], as also occurs in neurons [80] and other cell types [81–83] under stressful conditions.

**Fig. 8:**
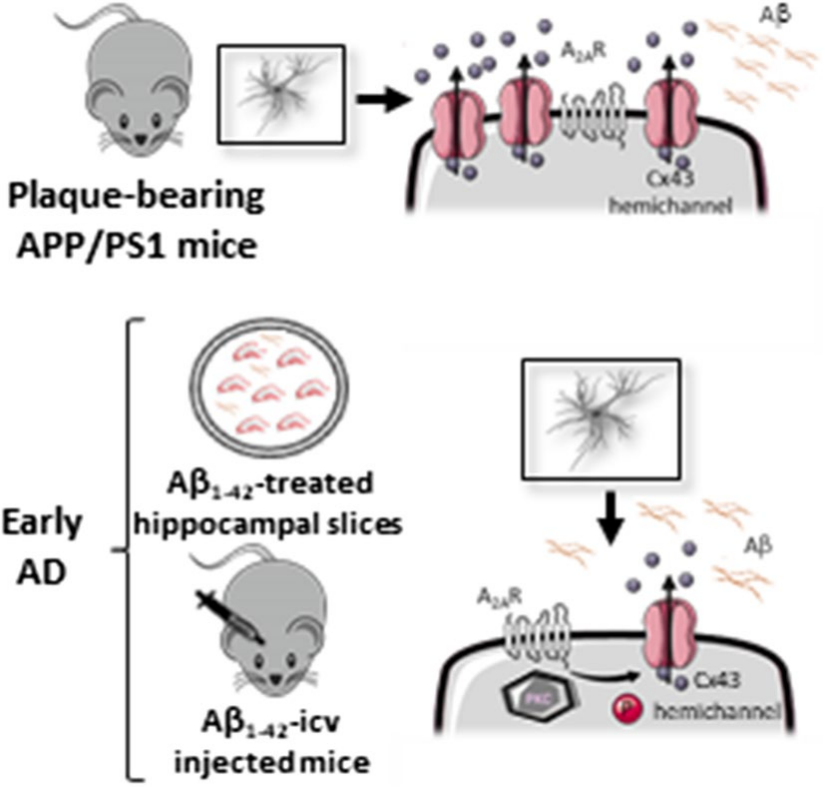
Hypothetical mechanistic interaction between Cx43 and A_2A_R in AD. In early and late stages of AD models, there is an enhancement in the activity of astroglial hemichannels. In early AD, the heighten activity of hemichannels seems to be due to an increase in Cx43 phosphorylation. Cx43 phosphorylation at Ser368 occurs through PKC, an event downstream of A_2A_R activation. On the other hand, in plaque-bearing APP/PS1 mice, the augment in astrocytic hemichannel activity is linked with an enhancement of Cx43 levels

In conclusion, the present study reinforces the role of astrocytic dysfunction in AD pathogenesis. In transgenic APP/PS1 mice displaying diffuse Aβ plaques, deficits in hippocampal-dependent memory were accompanied by alterations in astrocytic morphology and by an enhancement of astrocytic Cx43 hemichannel activity in the hippocampus. Similarly, models of early AD also showed an increase in the activity of Cx43 hemichannels involving an increase of Cx43 phosphorylation at Ser368. This increased activity of astrocytic Cx43 hemichannel activity in the hippocampus was regulated by astrocytic A_2A_R, as their genetic silencing increased both channels activity and Cx43 and phospho-Cx43 levels. Additionally, astrocytic A_2A_R also modulate the impact of Aβ on Cx43 hemichannel activity through a PKC-dependent mechanism (Fig. 8). Overall, these findings re-enforce the contention that astrocytic A_2A_R modulate astrocyte-to-neuron communication, which is altered in AD-like conditions. This may contribute to the benefits afforded by A_2A_R antagonists in early AD.

### Supplementary Information

Below is the link to the electronic supplementary material.

Supplementary file1 (DOCX 403 KB)

## Data Availability

The data that support the findings of this study are available from the corresponding author upon reasonable request.
