# Supplementary material for: Modification of astrocytic Cx43 hemichannel activity in animal models of AD: modulation by adenosine A2A receptors
Source: Cell Mol Life Sci. 2023 Oct 29;80(11):340. doi: 10.1007/s00018-023-04983-6 (PMC10613596; doi:10.1007/s00018-023-04983-6)
Supplement: Supplementary file 1 — Supplementary file1 (DOCX 403 KB) [file 18_2023_4983_MOESM1_ESM.docx]

**Supplementary data**


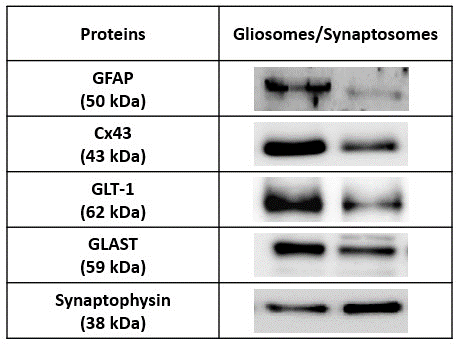


**Figure S1 - Validation of the purity of gliosomal preparations obtained from hippocampus of adult wild-type mice.** Comparison by Western blot analysis of the relative density of different astroglial markers (GFAP, Cx43, GLT-I and GLAST) and nerve terminal markers (synaptophysin) between hippocampal gliosomes and synaptosomes.


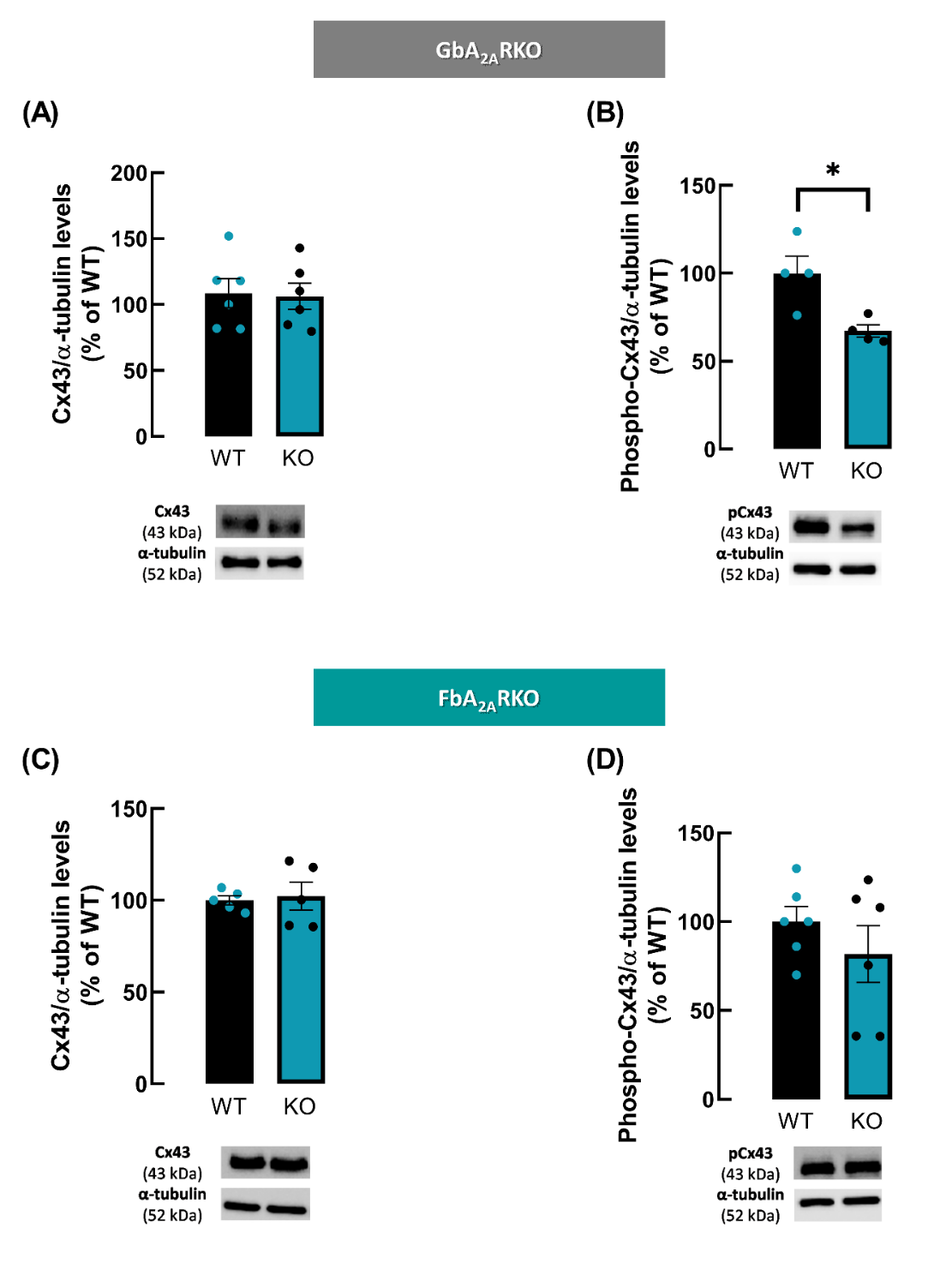


**Figure S2- Global A_2A_R deletion led to a decrease in phospho-Cx43 levels, whereas no alterations in either Cx43 or phospho-Cx43 were detected in FbA_2A_R-KO mice.** **(A)** In gliosomes (membranes from astrocytic processes) obtained from hippocampal tissue of GbA_2A_R-KO mice, Cx43 levels were unaffected, whereas the phospho-Cx43 levels were decreased relatively to WT **(B). (C)** In FbA_2A_R-KO mice, no alterations were observed in Cx43 or **(D)** in phospho-Cx43 levels. Ratio between Cx43 or phospho-Cx43 immunoreactivity to α-tubulin was expressed as percentage of WT. Data are mean ± SEM of 4-6 independent experiments. *p<0.05, unpaired Student’s *t*-test. Representative immunoblots for Cx43, phospho-Cx43 and α-tubulin are shown.

**
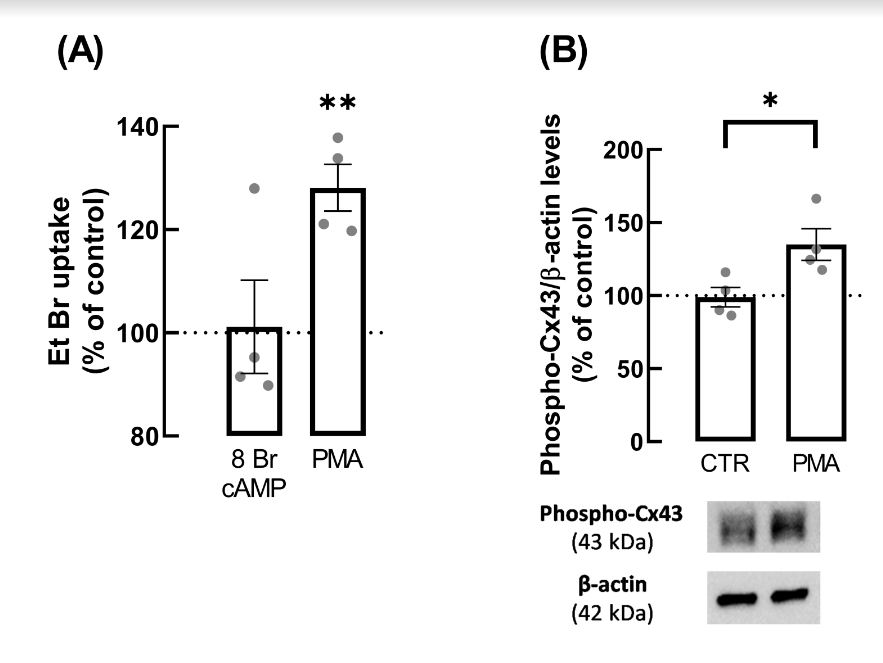
**

**Figure S3- PKC activation, but not PKA, increased hemichannels activity through Cx43 phosphorylation at Ser368 in astrocytic cultures. (A)** PKA activation with the cAMP analog, 8-Br-cAMP, had no effect on EtBr uptake in primary cultures of astrocytes, whereas PKC activation with the activator PMA significantly increased EtBr uptake. Cultured astrocytes were exposed to 8-Br-cAMP (5 µM) or to PMA (10 ng/mL) for 24 h. The activity of hemichannels was assessed through the mean fluorescent intensity of retained EtBr in the nucleus upon the subtraction of background values. Data are presented as percentage values relative to non-treated control cells (100%) and are mean ± SEM of 4 independent experiments **p < 0.0541 *vs*. control cells (100%), one sample t-test comparing with the hypothetical value of 100%. **(B)** PKC activation with PMA increased the levels of Cx43 phosphorylated at Ser368 in comparison with non-treated controls. Data are mean ± SEM of 4 independent experiments, presented as ratio between phospho-Cx43 immunoreactivity to β-actin and expressed as percentage of CTR. *p<0.05, unpaired Student’s *t*-test. Representative immunoblots for phospho-Cx43 and β-actin are shown below the average bar graphs.
